# Genome-wide association study meta-analysis uncovers novel genetic variants associated with olfactory dysfunction

**DOI:** 10.1186/s12863-025-01360-z

**Published:** 2025-09-17

**Authors:** Mohammed Aslam Imtiaz, Konstantinos Melas, Adrienne Tin, Valentina Talevi, Honglei Chen, Myriam Fornage, Srishti Shrestha, Martin Gögele, David Emmert, Cristian Pattaro, Peter Pramstaller, Franz Förster, Katrin Horn, Thomas H. Mosley, Christian Fuchsberger, Markus Scholz, Monique M.B. Breteler, N. Ahmad Aziz

**Affiliations:** 1 Population Health Sciences, German Centre for Neurodegenerative Diseases (DZNE), Venusberg-Campus 1/99, Bonn, 53127 Germany; 2 Department of Medicine, University of Mississippi Medical Center, Jackson, MS 39216 USA; 3 Department of Epidemiology and Biostatistics, Michigan State University, Michigan, USA; 4 Brown Foundation Institute of Molecular Medicine, McGovern Medical School, University of Texas Health Science at Houston, Houston, TX 77030 USA; 5 Human Genetics Center, School of Public Health, University of Texas Health Science at Houston, Houston, TX 77030 USA; 6 The Memory Impairment Neurodegenerative Dementia (MIND) Research Center, University of Mississippi Medical Center, Jackson, MS 39216 USA; 7 Eurac Research, Institute for Biomedicine, Via Volta 21, Bolzano, 39100 Italy; 8 Institute for Medical Informatics, Statistics and Epidemiology (IMISE), Medical Faculty, Leipzig University, Leipzig, Germany; 9 LIFE Research Center for Civilization Diseases, Medical Faculty, Leipzig University, Leipzig, Germany; 10 Institute for Medical Biometry, Informatics and Epidemiology (IMBIE), Faculty of Medicine, University of Bonn, Bonn, Germany; 11 Department of Neurology, Faculty of Medicine, University of Bonn, Bonn, Germany

**Keywords:** Genome-wide association meta-analysis, Olfactory dysfunction, Sense of smell, Odor identification test, Gene-mapping, PheWAS, Biochemical, Anthropometric, Two-sample MR

## Abstract

**Background:**

Olfactory dysfunction is among the earliest signs of many age-related neurodegenerative diseases and has been associated with increased mortality in older adults; however, its genetic basis remains largely unknown. Therefore, here we aimed to elucidate its genetic architecture through a genome-wide association study meta-analysis (GWMA).

**Methods:**

This GWMA included the participants of European ancestry (*N* = 22,730) enrolled in four different large population-based studies followed by a multi-ancestry GWMA including participants of African ancestry (*N* = 1,030). Olfactory dysfunction was assessed using a 12-item smell identification test.

**Results:**

GWMA revealed a novel genome-wide significant locus (tagged by single nucleotide polymorphism rs11228623 at the 11q12 locus) associated with olfactory dysfunction. Gene-based analysis revealed a high enrichment for olfactory receptor genes in this region. Phenome-wide association studies demonstrated associations between genetic variants related to olfactory dysfunction and blood cell counts, kidney function, skeletal muscle mass, cholesterol levels and cardiovascular disease. Using individual-level data, we also confirmed and quantified the strength of these associations on a phenotypic level. Moreover, employing two-sample Mendelian Randomization analyses, we found evidence for causal associations between olfactory dysfunction and these phenotypes.

**Conclusions:**

Our findings provide novel insights into the genetic architecture of the sense of smell and highlight its importance for many aspects of human health. Moreover, these findings could facilitate the identification and monitoring of individuals at increased risk of olfactory dysfunction and associated diseases.

**Supplementary Information:**

The online version contains supplementary material available at 10.1186/s12863-025-01360-z.

## Background

Olfactory function is paramount to both safety and quality of life, enabling detection of hazardous or unpleasant odors and contributing to the enjoyment of scents, food and drink. Impairment of olfaction is very common, affecting approximately 1 in 5 adults with an increased prevalence among older individuals [24]. Indeed, aging is a major determinant of olfactory dysfunction and is thought to affect both the central and peripheral olfactory system. Other risk factors associated with smell loss in adults include sinonasal diseases, smoking, and alcohol consumption [24]. Moreover, reduced sense of smell is a well-established consequence of coronavirus disease 2019 and one of the earliest markers of many neurodegenerative diseases [25, 27, 36]. Importantly, olfactory dysfunction itself has been suggested as a risk factor associated with cognitive decline [22], frailty [40], cardiovascular diseases [35], kidney function [44], and increased mortality [26]. However, the causality of these associations remains to be elucidated. Thus, uncovering the genetic architecture of olfactory dysfunction could not only provide novel molecular targets for its treatment, but could also be instrumental to assessing whether decreased sense of smell is causally related to adverse health outcomes.

Despite the high prevalence of olfactory dysfunction and its involvement in a variety of diseases, the genetic architecture of olfactory dysfunction remains largely unknown. Odor identification, the most commonly studied component of olfactory function, has been shown to have a low to moderate heritability [13, 23]. A previous genome-wide association study (GWAS) of olfactory function identified nine genome-wide significant loci associated with odor identification among African Americans (*N* = 1,979), but only two among European Americans (*N* = 6,582).^10,11^ Interestingly, many of these regions were related to neurodegenerative and neuropsychiatric diseases [10, 11]. More recently, Raj et al. examined the association between single nucleotide polymorphisms (SNPs) located in or near olfactory receptor genes (32,282 SNPs) and the ability to identify individual odors, detecting a larger number of SNPs (9,267 SNPs) at a suggestive statistical significance level (*p* < 0.001). However, none of these SNPs remained significant after adjustment for multiple testing, failing to replicate the findings from the previous GWAS [33]. 

Considering the critical role of olfactory dysfunction in many aspects of human health, here we aimed to elucidate its genetic architecture by performing the largest GWAS and meta-analysis of sense of smell to date, among adults of European and African ancestry, using data from four different large-scale, population-based studies. Moreover, using a two-sample Mendelian Randomization (MR) approach, we investigated the causal relationship between olfactory dysfunction and different health-related outcomes.

## Methods

### Study population

We included 1,030 individuals of African American ancestry (AAs) from the Atherosclerosis Risk in Communities (ARIC) Study [47], and 22,730 individuals of European ancestry (EUR) from the Rhineland Study, the ARIC Study, the Leipzig Research Centre for Civilization Diseases (LIFE) -Adult-Study [12] and the Cooperative Health Research in South Tyrol (CHRIS) study [29], who had complete genetic and olfactory function data (Table 1). The Rhineland Study is an ongoing prospective cohort study enrolling individuals aged 30 or above from two geographically defined areas in Bonn, Germany. The only exclusion criterium is insufficient command of the German language required for providing informed consent. The ARIC Study is an ongoing longitudinal study that was established in 1987–1989 to investigate risk factors for cardiovascular diseases. The LIFE-Adult-Study is a population-based cohort study investigating the prevalence and incidence of common diseases and subclinical disease phenotypes, the complex interactions between genetic and lifestyle factors regarding the co-occurrence and development of subclinical phenotypes and diseases, and the role of biomarkers to predict disease initiation and progression. The study comprises an age- and sex-stratified random sample of 10,000 adult individuals (aged 18–79 years) from Leipzig, Germany. The Cooperative Health Research in South Tyrol (CHRIS) Study is an ongoing longitudinal study established in 2003 to investigate genetic and lifestyle determinants of health and healthy aging in a single administrative district of the alpine Bolzano-South Tyrol province of Italy with a stable population, cooperative administration, shared single reference hospital, relatively homogenous customs and environment, and high healthy life expectancy. 13 393 adults aged ≥ 18 years (median 46, range 18–94, 54% female) participated in a baseline visit at the district reference hospital in 2011–2018.

**Table 1:** Baseline characteristics of participating cohorts

Characteristic	RHINELAND	ARIC EA	ARIC AA	LIFE-ADULT	CHRIS
N	6580	3654	1030	4771	6696
Age (years)	55.9 ± 13.5	75.9 ± 5.2	75.0 ± 5.1	57.4 ± 12.97	44.6 ± 16.5
Women, %	55.4	56.0	66.0	51.4	53.2
Olfactory dysfunction score (mean ± SD)	2.10 ± 1.71	2.48 ± 2.29	4.01 ± 2.59	2.06 ± 1.72	1.6 ± 1.55
*APOE* ε4 allele carriers, %	1233 (26%)	922 (25%)	394 (38%)	1148 (24.1%)	2396 (35%)
Global cognition score (mean ± SD)	−0.57 ± 0.55	-	-		
MMSE (mean ± SD)	-	27.9 ± 2.3	25.6 ± 3.3		28.98 ± 1.62
Cognition (CERAD)				24 ± 6.34	

*AA* African-American, *ARIC* Atherosclerosis Risk in Community Study, *CHRIS* The Cooperative Health Research in South Tyrol, *EA* European-American, *EUR* European, *LIFE-Adult* LIFE-Adult Cohort, *MMSE* Mini-Mental State Examination, range of possible score 0–30, *CERAD* Consortium to Establish a Registry for Alzheimer’s disease Neuropsychological Battery, *RHINELAND* Rhineland Study, *SD* Standard deviation

### Assessment of olfactory function

In the Rhineland Study, the ARIC Study and the LIFE-Adult-Study, olfactory function was assessed using the 12-item “Sniffin’ Sticks” odor identification test (SIT-12), a widely utilized screening instrument for assessing odor identification ability [21]. This is assessed by using twelve felt-tip sticks from a test kit (Burghart Messtechnik GmbH, Germany), each carrying a distinct odorant. These sticks were consecutively positioned approximately 2 cm in front of both nostrils for 3 to 4 seconds by trained technicians in a well-ventilated room. Participants were then asked to choose only one out of four answer options for each odorant. The time interval between two consecutive odor presentations was at least 20 seconds. In the CHRIS study, the 16-item “Sniffin’ Sticks” odor identification test was employed and for this analysis restricted to the SIT-12 items, where results from the four pens *(turpentine*, *garlic*, *apple*, and *anise*) were excluded to make the assessment of olfactory dysfunction across all cohorts comparable. Olfactory dysfunction was defined as the total number of incorrectly identified odors on the SIT-12 test (range 0–12**)**.

### Genotyping, quality control and imputation

Genotyping was performed in all four cohorts using commercially available genetic arrays followed by standard quality control measures. In brief, for the meta-analysis, quality control was performed using PLINK (version 1.9), excluding SNPs based on poor genotyping rate (< 99%), minor allele frequency (MAF) < 1%^3^ or Hardy-Weinberg Disequilibrium (HWE) (*p* < 1 × 10^−6^). Imputation of genotypes was performed through IMPUTE (version 2) [20], using as reference panels 1000 Genomes phase 3 version 5 in the Rhineland Study and the LIFE-Adult cohort, 1000 Genomes version 1 phase 3 in the ARIC Study [3]and TOPMed in the CHRIS cohort. The genetic variants were mapped to human genome coordinates based on GRCh37/hg19. Variants with imputation quality score below 0.3 were excluded [42]. In the ARIC study, genotyping was performed using the Affymetrix GeneChip SNP Array 6.0, applying standardized quality-control filters for call rate (< 95%), Hardy Weinberg equilibrium (HWE) (*p* < 1 × 10^−5^) for SNPs and for the samples to exclude individuals with call rate (< 95%) before imputation. In the Rhineland Study, blood samples were genotyped using the Illumina Omni-2.5 exome array and processed with GenomeStudio (version 2.0.5). Quality control was performed using PLINK (version 1.9). SNPs were excluded based on poor genotyping rate (< 99%) or HWE (*p* < 1 × 10^−6^). Additionally, participants with poor quality DNA samples were excluded because of poor call rate (< 95%) (*n* = 51), abnormal heterozygosity (*n* = 100), cryptic relatedness (*n* = 472) or sex mismatch (*n* = 43). To account for variation in the population structure, which may otherwise cause systematic differences in allele frequencies [31], EIGENSTRAT (version 16000) was used. EIGENSTRAT uses principal component analysis to detect and correct for population structure, which resulted in the exclusion of an additional 164 participants from non-European descent. Finally, imputation was performed using IMPUTE (version 2) [20] and the 1000 Genomes version 3 phase 5 as the reference panel [3]. In the LIFE-Adult study, the Affymetrix AXIOM-CEU 1 array was used for assessment of genotypes. Exclusion was based on low sample call rate (< 97%), mismatches of reported and genotyped sex, duplicated samples or samples with unresolved relatedness and ethnic outliers. Imputation was performed with IMPUTE2 (Version 2.3.2) after pre-phasing with SHAPEIT (v2.r837). For the imputation the following filter criteria were used: SNP call rate < 97%, HWE < 1 × 10^−6^, plate association < 1 × 10^−7^, monomorphic SNPs; SNPs violating criteria of Affymetrix cluster measures FLD, HetSO and HomRO were removed too. In the CHRIS study, genotyping was carried out for consenting participants in three batches using the Illumina OmniExpressExome chip (*n* = 5,882), the Illumina Human Omni2.5Exome chip (*n* = 4,887), and the Illumina-based OmniEURHD chip from Life & Brain GmbH, Bonn (*n* = 2,694). Initial processing and quality control of returned raw genotyping data was performed on each genotype batch independently at the time of acquisition using the Illumina GenomeStudio software package with additional quality control following established protocols similar to those described above. Subsequently batches were merged together consecutively, discarding variants not present on all array chips, and excluding samples with > 5% missingness. The genotype dataset consisted of approximately 579,000 variants from autosomal chromosomes in 12,834 samples. TOPMed imputation was carried out with the topmed-freeze5 panel [39] on the Michigan Imputation Server (1.7.1) [8], using minimac4-1.0.2 [14]. Excluding markers with imputation R2 < 0.3, the CHRIS TOPMed-imputed dataset contains 35,061,390 variants.

### Genome-wide association studies

We performed an ancestry-specific GWAS of olfactory dysfunction in each cohort separately, using Generalized Linear Mixed Model Association Tests (GMMAT) [5]. Since olfactory dysfunction was defined as a count variable and followed a Poisson distribution, we applied a log-link function to model the association of each SNP with olfactory dysfunction, using the score test for computational efficiency [5, 15]. Indeed, using extensive simulations under different genetic models, it has been demonstrated that for the analyses of count outcome data Poisson regression models not only had more statistical power, but also yielded fewer false positives [15]. For variants that were genome-wide significant (*p* < 5 × 10^−8^) based on the score test, we rerun the analyses while applying the Wald test to obtain estimates for the effect sizes and associated standard errors. In model 1, we adjusted for age, sex and the first 10 genetic principal components to account for population structure. In model 2, we additionally adjusted for *APOE* ε4 carrier/non-carrier status and global cognitive function as these factors have previously been associated with a poor sense of smell [10]. 

### Meta-analysis of genome-wide association studies

The score test- and the Wald test-based results from GMMAT were meta-analyzed using the sample size-weighted or the fixed effects inverse variance-weighted method, respectively, as implemented in the meta-analysis tool for genome-wide association scans (METAL) [46]. Additionally, we performed a multi-ancestry meta-analysis by combining GWAS results from both European and African ancestry participants using a random effects model as implemented in METASOFT [17]. The genome-wide statistical significance threshold was set at *p* < 5 × 10^−8^.

### Genomic risk loci

We used the Functional Mapping and Analysis of GWAS (FUMA) platform to identify genomic risk loci based on GRCh37/hg19 human genome coordinates [45]. Genome-wide significant SNPs in relatively high linkage disequilibrium (LD) (i.e., r^2^ ≥ 0.6) with nearby SNPs were used to define genomic risk loci, merging LD blocks of independently significant SNPs within 250 kb of each other into a single genomic locus. Within each genomic locus, we defined the *lead* SNPs as those SNPs that are independent of one another at r^2^ < 0.1, using the 1000 Genome Phase 3 reference panel.

#### Variant annotation and gene mapping

Based on the standard 10 kb gap between SNPs and genes, each lead SNP was individually mapped to the gene. Variant annotation for each locus was based on lead SNPs and candidate SNPs, defined as those SNPs in LD with the lead SNP within a window of 250 kb and nominally significant at *p* < 0.05. Functionally annotated SNPs were subsequently mapped to genes based on (i) physical position on the genome (positional mapping), (ii) expression quantitative trait loci (eQTL) associations (eQTL mapping), and (iii) 3D chromatin interactions (Hi-C).

#### Gene and gene-set analysis

Gene-based analyses were performed using MAGMA (Multi-marker Analysis of GenoMic Annotation**)** v1.6 as implemented in FUMA, using the Bonferroni method for multiple testing. Mapped genes from SNP2GENE were further investigated using the GENE2FUNC tool in FUMA, which through a hypergeometric test assesses pathway enrichment of mapped genes in the Molecular Signatures Database (MSigDB) gene sets.

#### Gene expression sequencing and analysis

In the Rhineland Study, total RNA sequencing was performed using the TruSeq stranded total RNA kit (Illumina) on a NovaSeq6000 instrument (Illumina). Genes with overall mean expression levels greater than 15 reads and expressed in at least 95% of the participants were considered for further analysis.

#### RNA sequencing and gene expression analysis in the Rhineland study

Blood samples were collected between 7:00 to 9:45 in the morning from an antecubital or dorsal hand vein. For RNA sequencing, samples were stored in PAXgene Blood RNA tubes (PreAnalytix/Qiagen). PAXgene Blood RNA Tubes were thawed and incubated at room temperature to increase RNA yields. Total RNA was isolated according to manufactures’ instructions using PAXgene Blood miRNA Kit and the automated purification protocol (PreAnalytix/Qiagen). Differential blood cell counts (erythrocytes, neutrophils, eosinophils, basophils, lymphocytes, monocytes and platelets) were performed at the Central Laboratory of the University Hospital in Bonn, using EDTA-whole blood samples on a hematological analyzer Sysmex XN9000. RNA integrity and quantity were evaluated using the tapestation RNA assay on a tapestation4200 instrument (both from Agilent). We used 750 ng of total RNA to generate NGS libraries for total RNA sequencing using the TruSeq stranded total RNA kit (Illumina) following manufacturer’s instructions with Ribo-Zero Globin reduction. We checked library size distribution via tapestation using D1000 on a Tapestation4200 instrument (Agilent) and quantified the libraries via Qubit HS dsDNA assay (Invitrogen). We clustered the libraries at 250 pM final clustering concentration on a NovaSeq6000 instrument using S2 v1 chemistry (Illumina) in XP mode and sequenced paired-end 2*50 cycles before demultiplexing using bcl2fastq2 v2.20. Quality control of the sequencing was evaluated through FastQC v0.11.9. Following the trimming of low-quality score reads through Trimmomatic v.0.39 software, sequencing reads were aligned using STAR v2.7.1 and the human reference genome GRCh38.p13 provided by Ensembl. The count matrix was generated with STAR–quantMode GeneCounts using the human gene annotation version GRCh38.101. Genes with overall mean expression greater than 15 reads and expressed in at least 95% of the participants were included for further analysis. Raw counts were normalized and transformed using the varianceStabilizingTransformation function from DESeq2 (v1.30.1). We extracted the expression levels of mapped genes from total RNA sequencing data available from a subset of Rhineland Study participants (*n* = 1985). In brief, multivariable linear regression was used to assess the association between z-transformed gene expression levels and olfactory dysfunction while adjusting for age, sex, *APOE* ε4 carrier status, the first 10 genetic principal components, global cognitive function and sequencing batch. Subsequently, we tested whether this association was modified by age by including an interaction term between age and gene expression levels. For genes with a significant age interaction, we performed the linear regression analysis after stratifying in age tertiles (i.e., 30–50, 50–62 and 62–95 years). The age tertiles were defined using a data-driven approach to achieve an approximately equal number of participants in each subgroup.

For SNPs identified in the GWAS meta-analysis and their mapped genes, functional validation of the association between SNP and gene expression was performed using multivariable linear regression. Gene expression was coded as the dependent variable and the number of effect alleles (0, 1 or 2) was coded as a numeric independent variable, controlling for age, sex, the first 10 genetic principal components and sequencing batch.

### Phenome-wide association studies

We used the Open Target Genetics platform [16] to perform phenome-wide association studies (PheWAS) for the systematic identification of phenotypes associated with genetic variations related to olfactory dysfunction. The Benjamini-Hochberg false discovery rate (FDR) method was used for multiple comparisons adjustment.

### Phenotypic associations using individual-level data

In addition, using individual-level data from the Rhineland Study, we assessed whether phenotypes associated with genetic variants of olfactory dysfunction (after FDR correction) were also associated with a poor sense of smell on a phenotypic level. Age, sex and smoking data were based on self-reports using questionnaires. Smoking was coded as a dichotomous variable (current vs. non-current smoker). Participants with missing data on smoking were classified as current smokers when their cotinine metabolic levels, measured with the Metabolon HD4 platform, exceeded the non-smoker sample-defined 97.5 percentile. Body mass index (BMI) was measured as weight (kg) divided by height squared (m [2]). Skeletal muscle mass (kg) was derived from bioimpedance analysis. Muscular strength was measured using the hand-held Jamar Plus Digital Dynamometer (Patterson Medical, USA). The grip force of each hand was measured three times and the average of the hand grip strength was calculated. Hypertension was coded as “yes” in case of antihypertensive drug use or high blood pressure (mean systolic blood pressure > = 140 mmHg or diastolic blood pressure > = 90 mmHg), and as “no” otherwise. Heart rate was measured as number of heart beats per minute. Cardiovascular conditions including stroke, heart failure and coronary artery disease (CAD) were defined as self-reported physician diagnosis. Differential blood cell counts (e.g., erythrocytes, leukocytes, basophils, eosinophils, lymphocytes, monocytes, neutrophils) were measured at the Central Laboratory of the University Hospital in Bonn using EDTA-whole blood samples on a hematological analyzer Sysmex XN9000. Habitual dietary intake (ml/day) was assessed by a self-administered semi-quantitative food frequency questionnaire (FFQ). We assessed whether phenotypes associated with genetic variants of olfactory dysfunction (after FDR correction) were also associated with a poor sense of smell on a phenotypic level using multivariable regression models. Statistical significance was inferred at *p* < 0.05. We log-transformed white blood cell (WBC), eosinophil, neutrophil and lymphocyte cell counts, as well as skeletal muscle mass, to account for their skewed distributions. All the phenotypes were adjusted for poor sense of smell and further corrected for age and sex. We additionally adjusted for potential risk factors such as smoking when the dependent variable was heart rate, white blood cell counts for neutrophil, eosinophil and lymphocyte cell counts and BMI for skeletal muscle mass and cardiovascular diseases (CVD). All the numerical variables were standardized to a mean of 0 and a standard deviation of 1 to allow for better comparison of the effect sizes across different traits. To this end, we employed multivariable regression models with statistical significance inferred at FDR-adjusted *p* < 0.05.

### Two-sample Mendelian randomization

We employed a two-sample MR approach to test whether the associations between olfactory dysfunction and the phenotypes identified in the previous step were causal, using the TwoSample MR package [19]. For the outcomes, we obtained GWAS summary statistics using the IEU GWAS database (Supplementary Table 13) [19]. Specifically, we obtained the GWAS summary statistics for coffee intake [19], lymphocyte count [6], cystatin C levels [28], pulse rate [19], hypertension [9], appendicular lean mass [30], total cholesterol levels [37], cardiovascular disease [9], white blood cell count [43], lymphocyte percentage of white cells [2], neutrophil percentage of white cells [2], hand grip strength (right) [19], hand grip strength (left) [19], basophil count and neutrophil cell count [19]. As genetic instruments for the exposure we used genome-wide significant SNPs from the olfactory dysfunction GWAS meta-analysis. SNPs in LD were clumped based on r^2^ < 0.01 at a 10 Mb window before the MR analyses. To assess the risk of weak instrument bias, we calculated the F-statistic for the selected genetic instruments [38]. The Benjamini-Hochberg false discovery rate (FDR) method was used for multiple comparisons adjustment.

## Results

### Population characteristics

The study and ancestry specific population characteristics are provided in Table 1, Supplementary Table 1. On average, participants of European ancestry had a lower degree of olfactory dysfunction and scored higher on cognitive tests compared to those of African ancestry. Moreover, *APOE* ε4 allele carrier frequency was lower in participants of European ancestry.

### GWAS meta-analysis

**Fig. 1:**
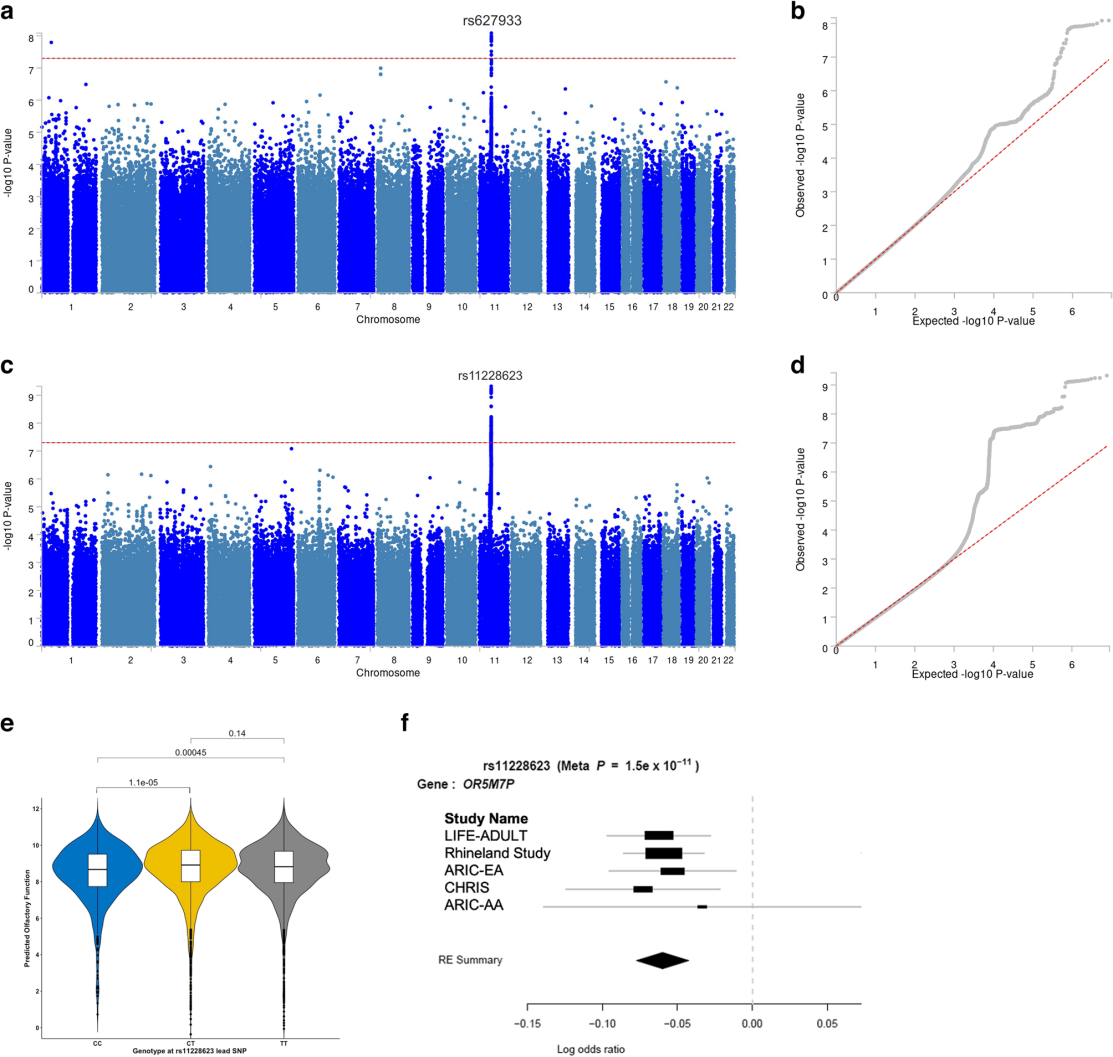
Genome-wide association meta-analysis of olfactory function. Manhattan (**a**) and corresponding quantile-quantile plot (**b**) of the genome-wide meta-analysis of sense of smell in people of European ancestry for model 1. Manhattan (**c**) and corresponding quantile-quantile plot (**d**) of the genome-wide meta-analysis of the sense of smell in people of European ancestry for model 2. The horizontal red dashed lines indicate the threshold for genome-wide significance (i.e., p < 5 × 10^−8^). **e** Box and violin plots showing adjusted olfactory function (i.e., score on the Sniffin’ Sticks odor identification test) for each genotype (CC, CT, or TT) based on variations in the lead SNP (rs11228623) in the Rhineland Study participants. Olfactory function was adjusted for age, sex, global cognitive ability, *APOE* ε4 carrier status, and the first 10 genetic principal components, by regressing out the effects of these covariates using a Poisson regression model. Between group comparisons were made using pairwise Wilcoxon rank-sum tests (the p-values are indicated in the plot for reach pairwise comparison). **f** Forest plot showing the specific effect of the lead SNP at the *OR5M7P* locus. Forest plots display the p-value of the lead SNP in METASOFT meta-analysis (Meta P) and the p-value, log(odds ratio) and its associated 95% confidence intervals (whiskers) of the lead SNP in the GWAS of olfactory dysfunction in each European and African cohort

**Fig. 2:**
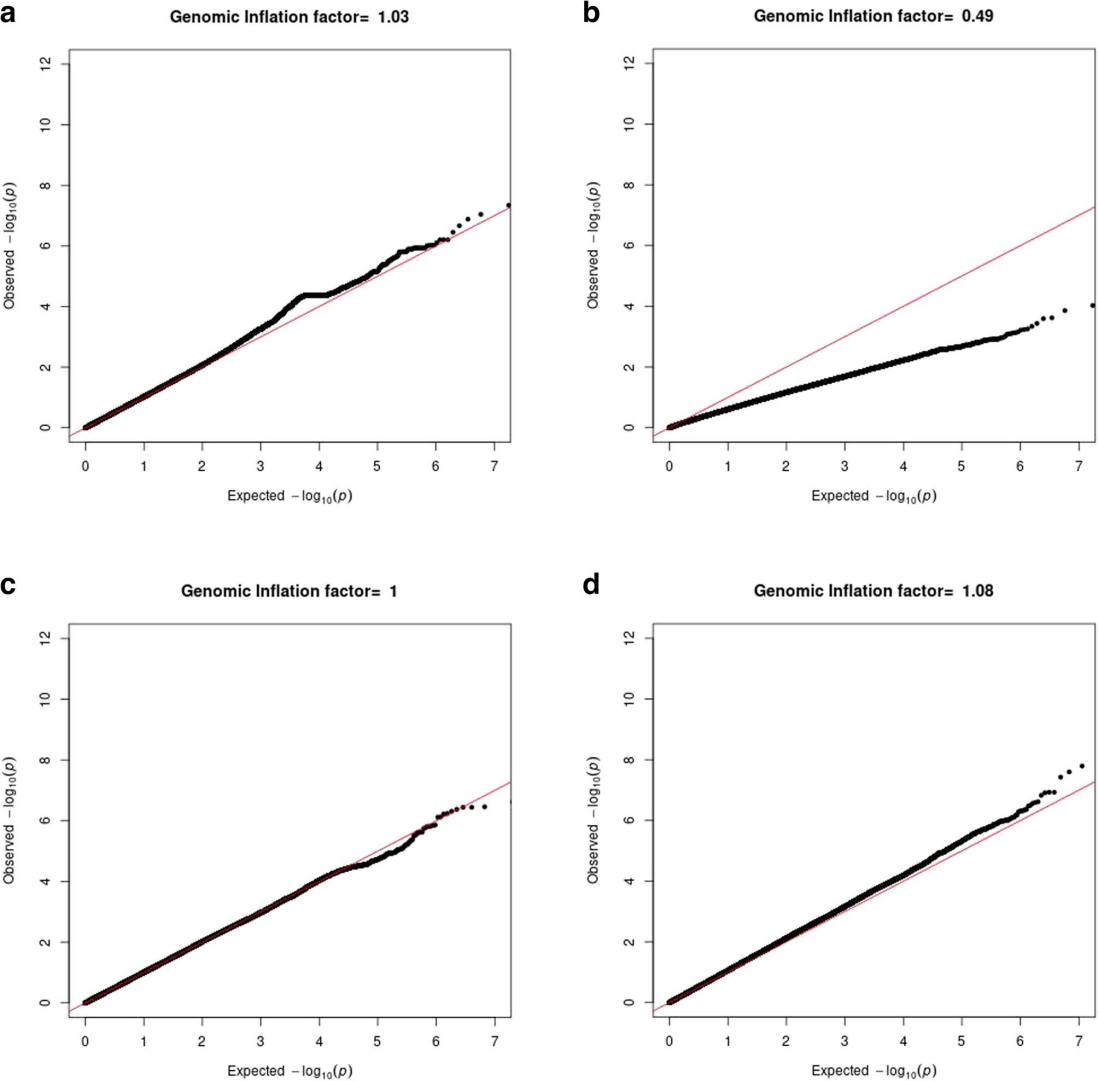
Quantile-quantile plots per cohort. Quantile-quantile plots for the GWAS-summary statistics on olfactory dysfunction in people of European ancestry in each cohort (**a)** Rhineland Study, **b** ARIC study, **c** LIFE-Adult study, and **d** CHRIS study in model 2

**Fig. 3:**
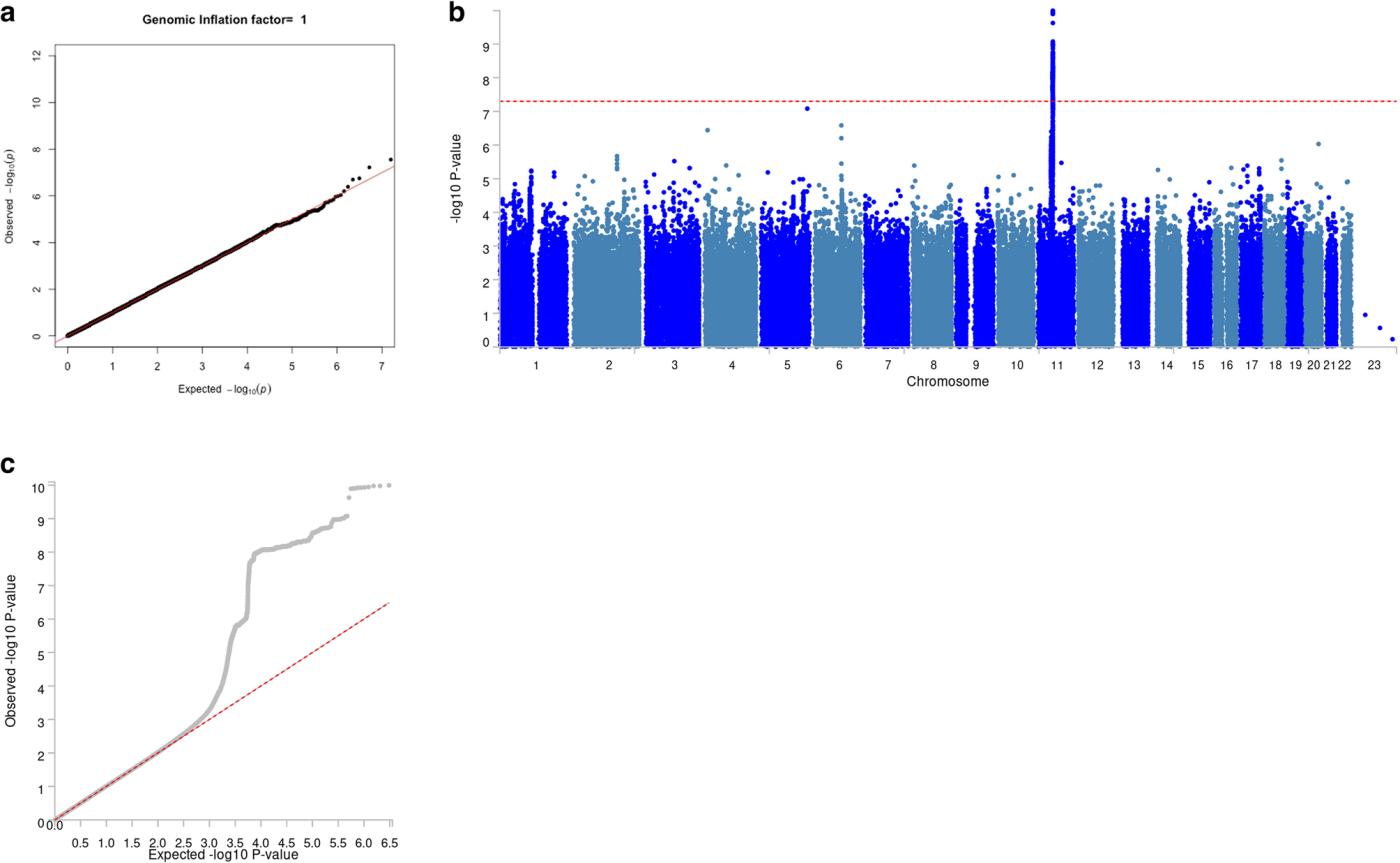
Sensitivity analysis using adjusted p-values. Quantile-quantile plot for the GWAS-summary statistics from model 2 in ARIC cohort using adjusted p-values (**a**). Manhattan plot (**b**) and corresponding quantile-quantile plot (**c**) of the genome-wide meta-analysis in the European ancestry participants after inclusion of the adjusted p-value summary statistics from the ARIC cohort. The horizontal red dashed line indicates the threshold for genome-wide significance (*p* < 5 × 10^−8^)

**Fig. 4:**
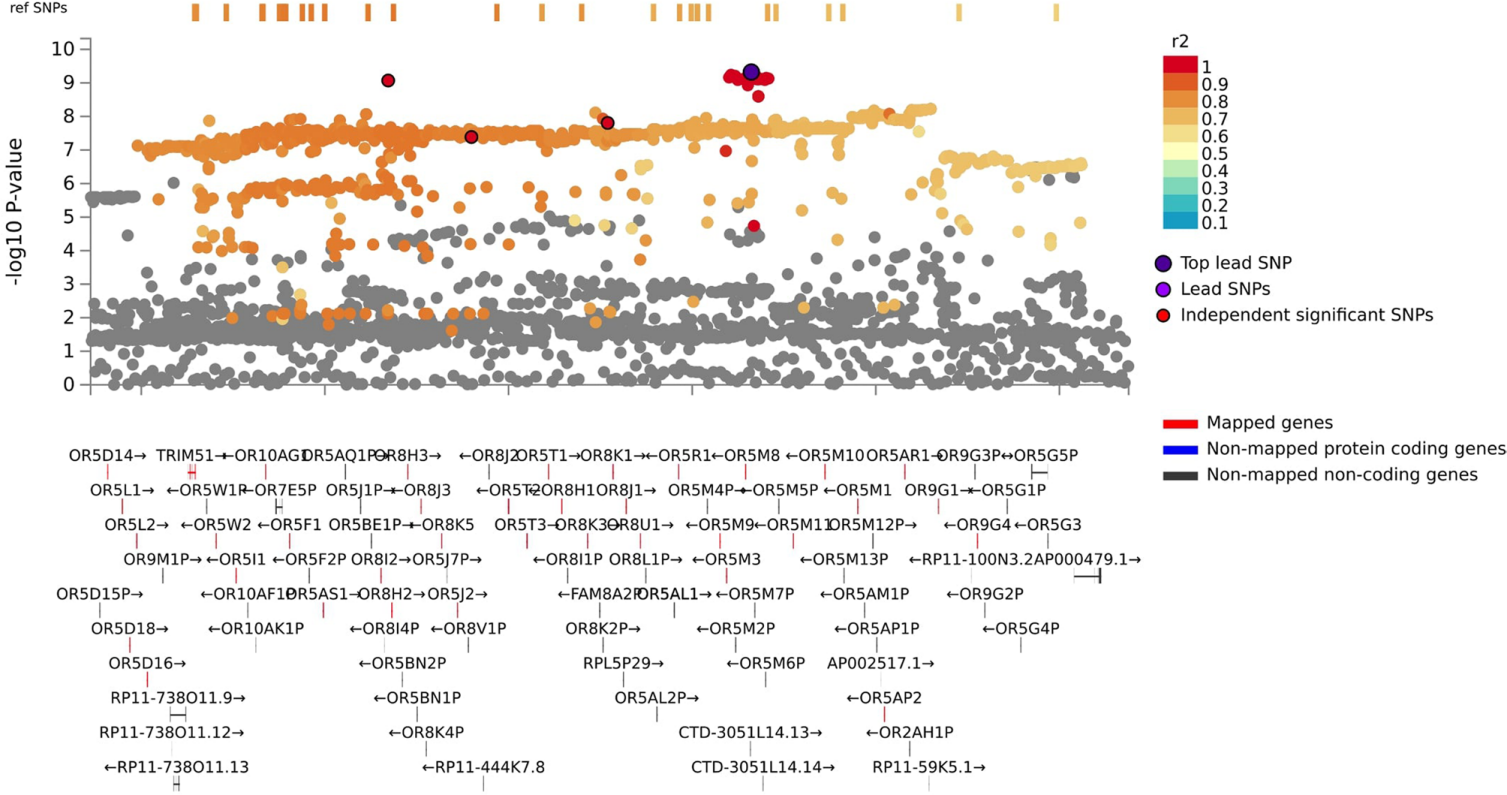
Regional plots of the lead and candidate genetic variants. The figure shows positional mapping of the 11q12 locus with the top lead single nucleotide polymorphism (SNP), as well as variants in linkage disequilibrium with this SNP according to the r [2]-color coded key

The GWAS meta-analysis of European ancestry participants identified 22 and 1523 genome-wide significant SNPs located on chromosome 11 based on results from model 1 and model 2 respectively, both pointing to the same genomic locus, 11q12, based on GRCh37/hg19 coordinates (Fig. 1). Overall, the genomic inflation factor (λ) in each European cohort was low, ranging from 0.49 t to 1.01 (Fig. 2). Because the λ-value was relatively low (0.49) in the ARIC European ancestry cohort, indicating genomic deflation, we also performed a sensitivity analysis in which we corrected the p-values in this group by dividing the chi-squared statistic by λ [1], and re-running the European-based meta-analysis. This, however, did not change the results (Fig. 3). In model 2, the meta-analysis identified one lead SNP rs11228623, as well as three independent significant SNPs (rs12786376, rs34099256, and rs369532258), across one genomic risk locus (11q12) (Table 2; Fig. 4, and Supplementary Tables 2–5). In the multi-ancestry GWAS meta-analysis, the associations of the lead SNP (rs11228623) and one of the independent SNPs (rs12786376) with olfactory dysfunction remained directionally consistent and genome-wide significant (Table 2).

**Table 2:** Independent genome-wide significant single nucleotide polymorphisms (r^2^ < 0.6 and *p* < 5 × 10^−8^) associated with olfactory dysfunction in European GWAS meta-analysis, and comparison with the cross-ancestry meta-analysis. The results displayed are summary statistics derived from the GWAS meta-analysis of Wald test results

SNP	Chr	Position (GrCh37)	Effect Allele	Other Allele	EAF	Location	Gene	Meta-analysis^b^					Cross-ancestry meta-analysis^†^		
								EUR					EUR & AFR		
								Beta	SE	*P*-value	Direction^c^	I2	Beta	SE	*P*-value
rs11228623^a^	11	56,264,453	T	C	0.6	Downstream	*OR5M7P*	−0.06	0.009	2.2 × 10^−11^	-,-,-,-	0	−0.05	0.008	1.5 × 10^−11^
rs12786376	11	55,868,951	A	C	0.8	intergenic	*OR8H2*	−0.09	0.015	8.5 × 10^−11^	-,-,-,-	0	−0.09	0.015	6.4 × 10^−11^
rs34099256	11	56,108,004	T	TA	0.5	intergenic	*OR8K2P*	0.06	0.011	9.3 × 10^−9^	+,?,+,?	0	-	-	-
rs369532258	11	55,959,646	T	TA	0.5	intergenic	*OR8V1P*	0.06	0.010	2.0 × 10^−8^	+,?,+,?	34.3	-	-	-

*AFR* African-American, *Chr* chromosome, *EUR* European, *SE* Standard Error, *EAF* Effect Allele Frequency, *I2* heterogeneity index (0-100 scale)

^a^The lead independent single nucleotide polymorphism of the genomic locus

^b^The meta-analyses were based on results from model 2, in which we adjusted for age, sex, APOE genotype, cognitive function and the first 10 genetic principal components

^c^Effect directions are shown in the order: Rhineland study, Atherosclerosis Risk in Community Study (ARIC); LIFE-Adult Cohort; Cooperative Health Research in South Tyrol (CHRIS) cohort

**Fig. 5:**
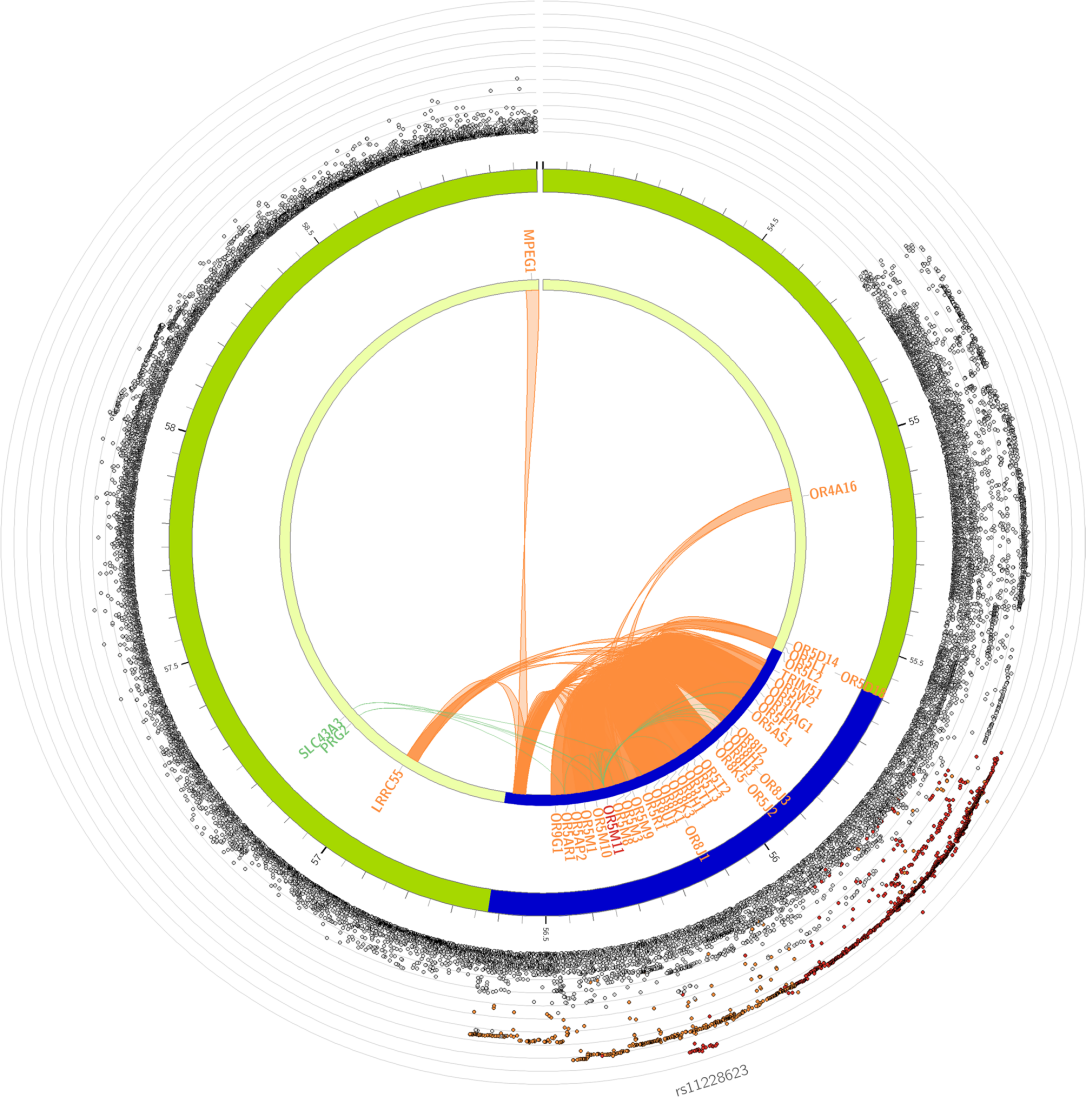
3D-chromatin interaction (Hi-C) mapping. Hi-C revealed significant interactions between genetic variants in *OR5M8* and other genes on chromosome 11 (FDR < 1 × 10^−6^), shown in orange

**Fig. 6:**
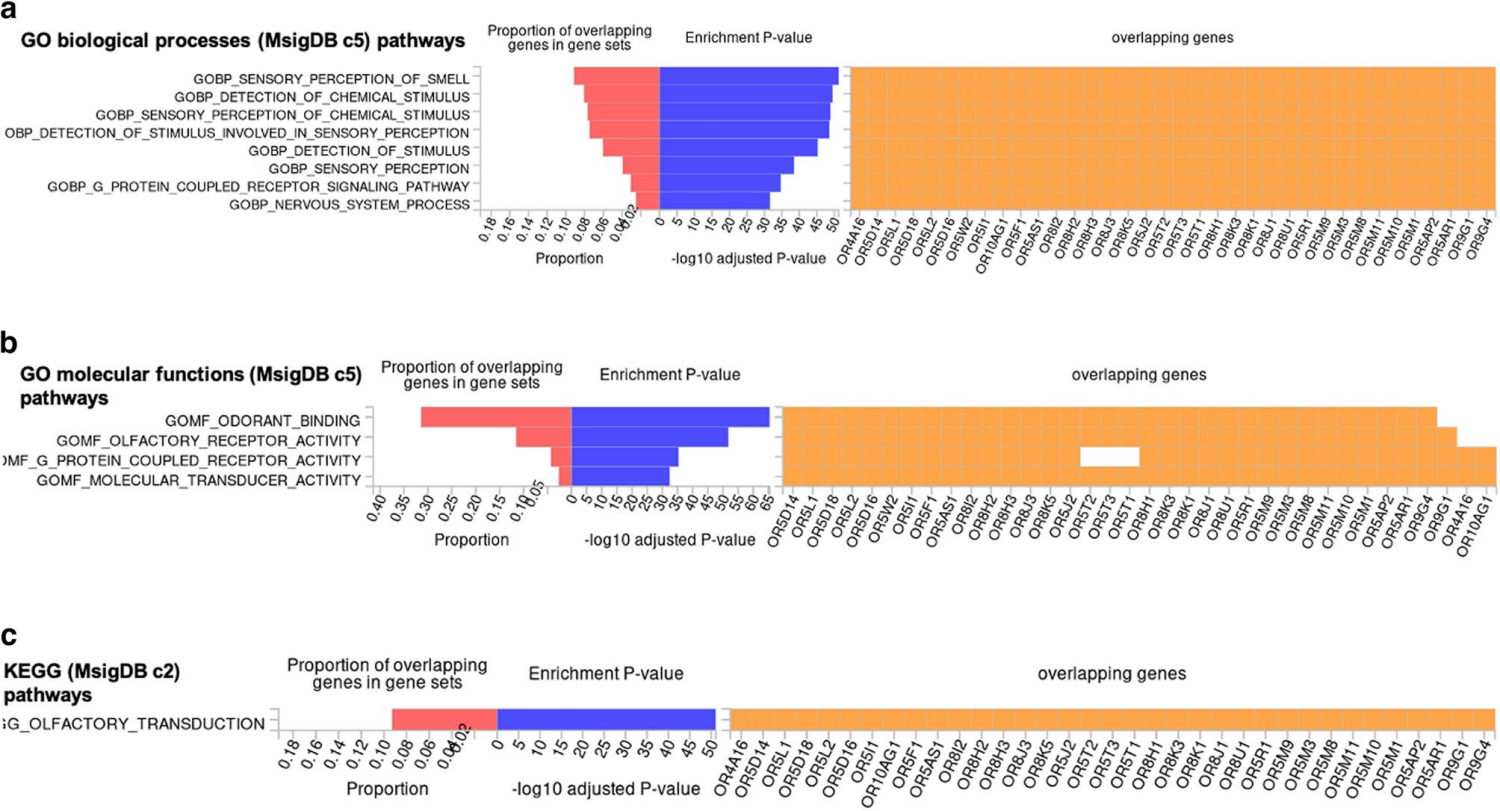
Gene-enrichment analysis. Enrichment analysis of mapped genes, according to Gene ontology (GO): biological process (**a**), GO: molecular functions (**b**), and Kyoto Encyclopedia of Genes and Genomes (KEGG) (**c**). Pathways and processes that are overrepresented in the gene set of interest are shown. Input genes that are overlapping in the pathway or process, the enrichment p − value and the proportion of the overlapping genes (input genes relative to the tested gene set) are shown

The olfactory dysfunction-associated SNPs were mapped to genes based on positional, eQTL and chromatin interaction mapping. Positional mapping identified 34 olfactory receptor genes. We discovered 3 genes (*OR5M11*,* SLC43A3* and *PRG2*) based on eQTL mapping of which one gene (*OR5M11*) overlapped with those identified through positional mapping (Supplementary Tables 6 & 7). Chromatin interaction mapping, based on Hi-C data, showed significant (FDR < 1 × 10^−6^) chromatin interactions between enhancers of candidate genes in this region and the promoter regions of *MPEG1*,* LRR45*, and *OR4A16*, as well as those of several other genes on chromosome 11q12 (Fig. 5 & Supplementary Table 8). After Bonferroni-correction, gene-based analysis using MAGMA identified 21 genome-wide significant (*p* < 2.6 × 10^−6^) genes, with *OR5M11* as the top hit (*p* < 8.4 × 10^−9^) (Supplementary Table 9). *OR5M11* is a protein coding gene that belongs to the family of olfactory receptors and is known to be involved in olfactory signaling pathways. In the MAGMA gene-set enrichment analysis, the top gene sets were enriched for “reactome hedgehog ligand biosynthesis” and “reactome degradation of beta catenin by the destruction complex” (Supplementary Table 10), but none survived multiple testing correction. Interrogation of MSigDB showed that the mapped genes at the 11q12 locus were significantly enriched for pathways related to “general odorant binding proteins, sensory perception of smell”, “molecular function, odorant binding” and “Grueneberg Ganglion, olfactory transduction” (Fig. 6).

### Gene expression analysis

Of the 41 genes identified through positional, eQTL and chromatin interaction mapping, expression levels were available for *MPEG1* (tagged by rs12786376) and *SLC43A3* (tagged by rs1811871001) in whole blood for 1985 participants in the Rhineland Study. Olfactory dysfunction was not associated with the expression levels of these two genes. However, a borderline significant interaction with age was found for *SLC43A3*. Age-stratified analysis showed that higher *SLC43A3* expression was associated with worse olfactory function at borderline significance for participants aged 30–50 years (Supplementary Table 11). However, we found no significant association between rs1811871001 and *SLC43A3* expression or rs12786376 and *MPEG1* expression.

### Phenome-wide association studies and phenotypic associations

**Fig. 7:**
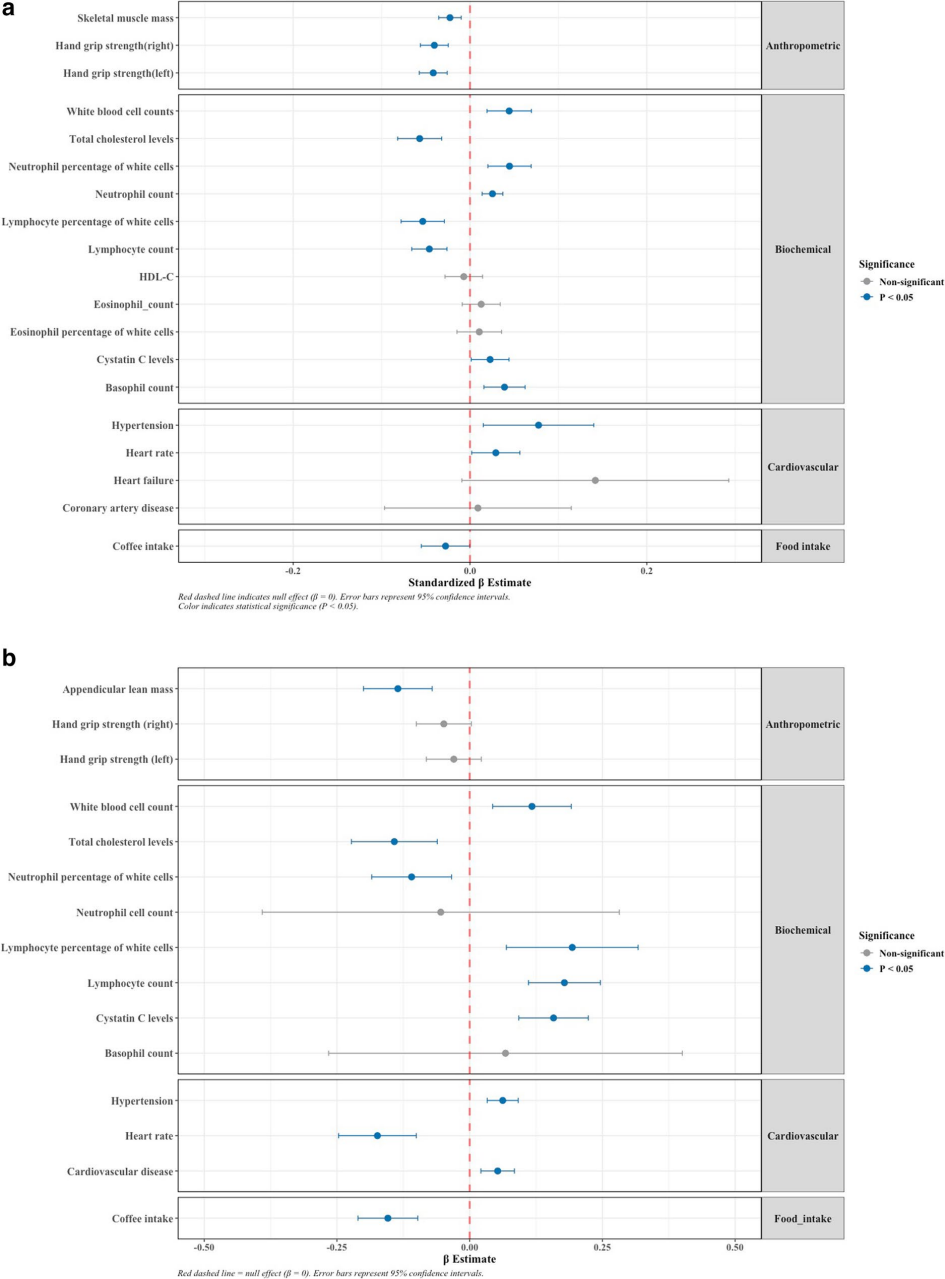
Comparison of phenotype-level and Mendelian Randomization estimates for associations between olfactory dysfunction and different traits and diseases identified through phenome-wide association studies. **a** Forest plot depicting associations between olfactory dysfunction and other phenotypes (identified through phenome-wide association studies after false discovery rate correction) using individual-level data from the Rhineland Study. The standardized regression estimate indicates the change in standard deviations in the outcome for one standard deviation increase in olfactory dysfunction. **b** Forest plot showing causal estimates from two-sample Mendelian Randomization analyses of the effect of olfactory dysfunction on other phenotypes (Wald ratio test). The regression estimate indicates the change in standard deviations in the outcome for the effect allele of the lead genetic variant (for binary outcomes, including hypertension, heart failure, coronary artery diseases, the regression estimate refers to the logarithm of the odds ratio)

The lead SNP (rs11228623), located within 5 kb downstream of the *OR5M7P* gene, was associated with 71 phenotypes after FDR correction (Supplementary Table 12). The identified traits included lymphocyte counts, eosinophil counts, lymphocyte percentage (%) of white cells, eosinophil percentage (%) of white cells, coffee intake, pulse rate, hypertension, mean appendicular mass and levels of cystatin-C (a marker of kidney function). In participants of the Rhineland Study, we could confirm that olfactory dysfunction was indeed significantly associated with lymphocyte, neutrophil and basophil cell counts, lymphocyte percentage of white blood cells, total white blood cell counts, coffee intake, skeletal muscle mass, hand grip strength, levels of cystatin-C, heart rate, hypertension, and at borderline significance with heart failure (*p* < 0.07) (Fig. 7A and Supplementary Table 13).

### Two-sample Mendelian randomization

After LD clumping, we identified one robust genetic instrument for olfactory dysfunction (rs11228623) with an F-statistic of 45.33, indicating a low probability of weak instrument bias. Two-sample MR analyses indicated causal associations between olfactory dysfunction and lymphocyte cell counts, as well as lymphocyte, neutrophil and eosinophil percentages of white blood cells, total white blood cell counts, appendicular lean mass, hand grip strength, coffee intake, hypertension, pulse rate and cardiovascular disease (Fig. 7B and Supplementary Table 14).

## Discussion

We performed the largest genome-wide meta-analysis of olfactory dysfunction to date (*N* = 22,730), discovering 1524 genome-wide significant variants and 21 genes associated with olfactory dysfunction in people of European descent. Importantly, the novel lead SNP (rs11228623-T at 11q12) was genome-wide significant and exhibited directionally consistent effects in both ancestry-stratified and multi-ancestry analyses. Gene mapping and gene set analysis prioritized multiple genes and pathways involved in odour reception and signalling. Importantly, combining PheWAS with individual-level and MR analyses, we found evidence for a causal association between olfactory dysfunction and several anthropometric, metabolic, cardiovascular, renal and inflammatory phenotypes.

We identified a genomic risk locus for olfactory dysfunction at 11q12, enriched for olfactory receptor genes related to sensory perception of smell and olfactory transduction. The lead SNP (rs11228623-T) at this region is located downstream of the *OR5M7P* pseudogene. Using eQTL analyses we mapped the independent SNP (rs12786376-A) to three other genes (*OR5M11*,* PRG2* and *SLC43A3)*. *OR5M11* is a protein-coding gene belonging to the superfamily of G-protein-coupled olfactory receptors, and was previously described as a contributing factor to the genetic burden underlying olfactory dysfunction [7]. Individual-level blood expression data were available for *SLC43A3*, and indicated an age-dependent association between *SLC43A3* expression levels and olfactory dysfunction. *SLC43A3* encodes a membrane transporter protein and has been shown to control free fatty acid flux in adipocytes [18]. To our knowledge, this is the first time this gene and its expression have been linked to olfactory dysfunction. Future mechanistic studies in model systems are warranted to replicate and functionally validate the associations between *SLC43A3* expression and olfactory dysfunction. The majority of the other identified genes are mainly expressed in the olfactory epithelium and, therefore, could not be detected in the blood transcriptome.

The results of our MR analyses indicate that olfactory dysfunction affects anthropometric, metabolic, cardiovascular, renal and inflammatory phenotypes, highlighting its detrimental effects across different organs and tissues. This included associations of olfactory dysfunction with skeletal muscle mass and hand grip strength, which have been identified before [32, 34]. A potential explanation could be that smell loss leads to changes in dietary habits, resulting in changes of muscle composition and strength. Conversely, it has been hypothesized that lifestyle factors, like exercise or comorbidities might concurrently affect muscle strength and the neuronal determinants of olfaction [34]. Our MR analyses support the former rather than the latter hypothesis. This is further supported by the causal association of olfactory dysfunction with coffee intake, a dietary habit and cholesterol levels, which are dependent on diet. Similarly, we found that olfactory dysfunction was causally associated with hypertension, increased heart rate and a higher prevalence of heart failure. This could indicate that olfaction affects cardiovascular risk through dietary patterns and obesity, while brain vascular damage or even cardiovascular medication may affect olfaction [4, 35]. Olfactory dysfunction was also causally associated with white blood cell counts and percentages, particularly those of neutrophiles and lymphocytes. As with anthropometric and cardiovascular phenotypes, this association could be mediated by dietary and/or metabolic changes. Alternatively, a neuro-immune interaction may be involved, since neurotransmitter release following olfactory stimuli might modulate the immune response to enhance defence against infections, for example when pathogens are detected by the olfactory receptors [41]. Perturbations of this neuro-immune cross-talk due to olfactory dysfunction may lead to changes in lymphocyte and neutrophil production.

The main limitation of our study is the relatively small number of participants from non-European ancestry; however, to the best of our knowledge, other large-scale population-based studies assessing olfactory dysfunction are currently lacking, precluding substantial increases of sample size in the near future. Moreover, we could replicate the association between the top genetic variant and olfactory dysfunction in people of European descent in those of African-American ancestry, but generalizability to other ethnic populations needs further investigation. Furthermore, assessing sex-specific differences in the genetic architecture of olfactory dysfunction would be valuable. Future studies with larger sample sizes are needed to detect potential sex-specific effects. Although in our MR analyses, we used a single SNP as an instrumental variable, the risk of horizontal pleiotropy is likely to be relatively low given the location of this variant in a region enriched for olfactory receptor genes. This was further supported by a high F-statistic for this variant, indicating a strong association between the genetic instrument and olfactory dysfunction, and thus low risk of weak instrument bias.

## Conclusions

We performed a multi-ancestry genome-wide meta-analysis of olfactory dysfunction in 22,730 individuals and found one genomic locus (11q12) robustly associated with olfactory dysfunction. Moreover, our analysis uncovered several genes such as *OR5M7P* and *OR5M11* related to olfactory dysfunction. Future studies employing perturbations of these genes in (animal) model systems are warranted for further functional validation and characterization of these findings. Importantly, we demonstrate that olfactory dysfunction is causally associated with muscle strength and mass, cardiovascular diseases, cholesterol levels, kidney function and white blood cell counts and composition. Thus, our findings provide new insights into the genetic architecture of olfaction and implicate olfactory dysfunction as a causal risk factor for anthropometric, metabolic, cardiovascular, renal and inflammatory phenotypes. Given the high prevalence of olfactory dysfunction among aging populations, the genetic variants and molecular pathways identified here could facilitate development of novel preventive and therapeutic strategies against a range of different age-associated diseases.

## Supplementary Information

Supplementary Material 1. Additional file 1: Table S1.Study characteristics and used software for the four studies used in this meta-analysis. Table S2. Genomic risk loci associated with olfactory dysfunction in model 1 and model 2Table S3**. **Lead SNPs associated with olfactory dysfunction in model 2**. **Table S4**. **Independent SNPs associated with olfactory dysfunction in model 2. Table S5**. **Genome-wide significant SNPs that are in LD (r2>0.6) with the independent SNPs in model 2. Table S6.ANNOVAR Annotation of the lead SNPs and SNPs that are in LD with lead SNPs in model 2. Table S7. Genes mapped based on positional, Eqtl and chromatin interaction mapping in model 2.Table S8.Chromatin interaction analysis of mapped genes in model 2.Table S9.MAGMA Gene-based analysis in model 2.Table S10.MAGMA Gene set analysis in model 2.Table S11. Functional validation of mapped genes using gene expression analysis.Table S12.PheWAS lookup of lead SNPs.Table S13.Associations between olfactory dysfunction and other phenotypes using individual-level data from the Rhineland Study.Table S14.Two-sample Mendelian Randomization analyses of the effect of olfactory dysfunction on other phenotypes

## Data Availability

Baseline characteristics of each cohort were assessed and summarized. Statistical and genetical analyses were conducted using command line tools such as Plink (version 1.9, [https://www.coggenomics.org/plink/](https:/www.coggenomics.org/plink)), R (version 4.0.4, [https://www.r-project.org/](https:/www.r-project.org)) and GMMAT (version 1.4.0, https://cran.r-project.org/web/packages/GMMAT/index.html). Meta-analysis of GWAS summary statistics was performed using METAL (latest version released on 2011-3-25, [https://csg.sph.umich.edu/abecasis/metal/download/](https:/csg.sph.umich.edu/abecasis/metal/download)). We used the web-based tool FUMA (version 1.3.8 and 1.5.2, https://fuma.ctglab. nl/) to perform LD pruning, lead loci detection and eQTL (GTEX v8, https://gtexportal.org/home/) analysis. MR analysis was performed using TwoSampleMR (version 0.5.7, https://mrcieu.github.io/TwoSampleMR/articles/introduction.html). The Rhineland Study’s data on which this manuscript was based, are not publicly available due to data protection regulations. Access to data can be provided to scientists in accordance with the Rhineland Study’s Data Use and Access Policy. Requests for additional information and/or access to the datasets can be send to [RS-DUAC@dzne.de](mailto: RS-DUAC@dzne.de). Data of LIFE-Adult are available in the framework of project agreements based on written requests. CHRIS data can be requested for research purposes by submitting a dedicated request to the CHRIS Access Committee ([access.request.biomedicine@eurac.edu](mailto: access.request.biomedicine@eurac.edu)). All authors had full access to the data and take responsibility for the integrity of the data as well as the accuracy of the analysis. All data generated during this study are included in this article and its supplementary information files. Summary statistics can be provided upon request. We used publicly available data in this manuscript, including data from GTEx version 8 (https://gtexportal.org/home/) for eQTL analysis and publicly available GWAS summary statistics (**Supplementary Table 13**) for TwoSample MR analysis. Summary statistics of the GWAS are publicly available through the GWAS Catalog (accession numbers GCST90668092 (model 1) and GCST90668093 (model 2)).

## Abbreviations

ARIC: Atherosclerosis Risk in Communities
CHRIS: Cooperative Health Research in South Tyrol
LIFE: Leipzig Research Centre for Civilization Diseases
SNP: Single Nucleotide Polymorphism
GWAS: Genome Wide Association Study
GWMA: Genome-Wide Association Study Meta-Analysis
GMMAT: Generalized Linear Mixed Model Association Tests
METAL: meta-analysis tool for genome-wide association scans
FUMA: Functional Mapping and Analysis of GWAS
MAGMA: Multi-marker Analysis of GenoMic Annotation
PheWAS: phenome-wide association studies
MR: Mendelian Randomization
MSigDB: Molecular Signatures Database
eQTL: expression Quantitative Trait Loci
